# Robust unified Granger causality analysis: a normalized maximum likelihood form

**DOI:** 10.1186/s40708-021-00136-2

**Published:** 2021-08-06

**Authors:** Zhenghui Hu, Fei Li, Minjia Cheng, Junhui Shui, Yituo Tang, Qiang Lin

**Affiliations:** 1 College of Science, Zhejiang University of Technology, Hangzhou, China; 2 College of Information Engineering, Zhejiang University of Technology, Hangzhou, China

**Keywords:** Unified Granger causality analysis, Normalized maximum likelihood, Inherent redundancy, Granger causality analysis, FMRI

## Abstract

Unified Granger causality analysis (uGCA) alters conventional two-stage Granger causality analysis into a unified code-length guided framework. We have presented several forms of uGCA methods to investigate causal connectivities, and different forms of uGCA have their own characteristics, which capable of approaching the ground truth networks well in their suitable contexts. In this paper, we considered comparing these several forms of uGCA in detail, then recommend a relatively more robust uGCA method among them, uGCA-NML, to reply to more general scenarios. Then, we clarified the distinguished advantages of uGCA-NML in a synthetic 6-node network. Moreover, uGCA-NML presented its good robustness in mental arithmetic experiments, which identified a stable similarity among causal networks under visual/auditory stimulus. Whereas, due to its commendable stability and accuracy, uGCA-NML will be a prior choice in this unified causal investigation paradigm.

## Introduction

Granger causality analysis (GCA) [1, 2], as a statistical predicting tool, provided causal descriptive relationships of candidate events in a sense of extra residual of regression comparing. Original GCA only describes the information flows between variables mathematically, which is predictive and may not truly describe the underlying causal relationships between events in a strict philosophic sense. However, due to its simple form of data-driven causal discovery paradigm, GCA has been widely applied and developed after it w as introduced into brain science. Considering the limitation of conventional GCA research paradigm, we proposed a unified paradigm of uGCA to investigate causal networks in the brain [3, 4]. This unified causal investigation paradigm is based on the category of code length to guide causal discovery, and then with the help of the principle of the minimum description length (MDL) principle to guide the generalized model selection of the whole process. Unified mathematical theory, no subjective choice of confidence level, and free comparison of candidate models make uGCA more advantageous.

Till now, we have extended several forms of uGCA behind introducing the crude two-part form, which actually is formalized upon different mathematical theories. The uGCA-TP form deriving by a two-part coding scheme, which to describe the fitting error term and model complexity term, behaved such as a Lagrange duality solving procedure. On the other hand, specifying some priors to its parameter space, the uGCA-MIX form adapts to behave such as a Bayes estimator, a simple approximation to this model selection issue is applied to derive the stochastic information criteria (SIC). In earlier two-part codes, it still remains some inherent redundancy. Thus the normalized maximum likelihood (NML) form of MDL, taking into account Fisher information, was developed based on the coding scheme of Shtarkov [5, 6]. In general, NML form restricted the early second part description of two-part MDL into a data space identified by parameter estimation [7]. This scheme for the generic model selection was formally introduced by Rissanen in 1996 and discussed its association with minimax theory. A sharper description length as the stochastic complexity and the associated universal process is derived for a class of parametric processes [8]. In addition, this description form is motivated by the maximum-likelihood estimate (MLE) which requires satisfying the Central Limit Theorem (CLT) [6, 9]. In this light, the associated uGCA-NML seems to be a more sensible choice, which not only eliminates the inherent redundancy in the coding process but also releases the priors to describe parameter space.

In previous studies, we focused on demonstrating the advantages of the uGCA paradigm over the conventional GCA paradigm. Although the characteristics of several different forms of uGCA had been described [4], we did not make a choice between them. In this study, we conclude that uGCA-NML will be a better selection for the most causal investigations. Not only for the advantages mentioned above, but for most of the current scientific researches which all tend to follow the research convention of larger samples and bigger data, these will yield to the requirements of uGCA-NML regarding the CLT to the more extent. At the same time, we consider that uGCA-NML is more consistent with our original intention of investigating causality based on a unified mathematical principle, and this form can more precisely incorporate the generalized model selection issues into the code length guided framework.

The rest of the article is organized as follows. In Sect. 2, we first briefly demonstrate the code length guided causal investigation paradigm. Then the uGCA-NML, deriving from the NML form, has been stated in detail, its generalized formulas also have been derived within a general model class. Immediately, the formula of description length guided causal investigation in an ordinary linear model is yielded out. In Sect. 3, we illustrate its advantages over other uGCA forms in 6-node network synthetic experiments. More importantly, in a task-related fMRI data set, uGCA-NML methods identified the consistent and more stable results of causality investigation of mental arithmetic networks under different stimuli. Sections  4 and 5 demonstrate comparisons among several forms from a mathematical modeling standpoint, and discuss its following potential development.

## Methods

Initially, we attempt to integrate the whole process of causal discovery into a unified mathematical theoretical framework. Inspired by the development of current coding theory and general computer theory, we consider incorporating the generalized model selection issues of GCA into the same benchmark, from which a unified code length guided causal investigation paradigm has emerged. At the same time, derived from information theory and stochastic complexity, the MDL principle has presented a systematic solution to the optimization problem of generalized model selection, and has different forms to cope with the diversity of data sources. Consequently, we developed the uGCA paradigm to explore causal relationships based on code length by means of the MDL principle.

### Description length guided causal investigation

Considering two variables, ${X_{N}}$ and ${Y_{N}}$, the description models associating with ${X_{N}}$ represent as

1 $$\begin{aligned} {\left\{ \begin{array}{ll} X_{t}=\sum _{j=1}^{n1}a_{1i}X_{t-j}+\epsilon _{1t}\\ X_{t}=\sum _{j=1}^{n2}a_{2i}X_{t-j}+\sum _{j=1}^{n3}b_{2i}Y_{t-j}+\epsilon _{2t}, \end{array}\right. } \end{aligned}$$

where $\epsilon _{t}$ is fitting residual. Distilling the concept of GCA paradigm, causal effect from *Y* to *X* within uGCA paradigm is defined by

2 $$\begin{aligned} \begin{aligned} F_{Y\rightarrow X}=L_{X}-L_{X+Y}, \end{aligned} \end{aligned}$$

where $L_{X}$ denotes the shortest coding length of restricted model in Eq. (1), and $L_{X+Y}$ denotes the shortest coding length of unrestricted model in Eq. (1) after adding $Y_{N}$. Causal effect from *Y* to *X* existed when $F_{Y\rightarrow X} >0$, or else no causal effect existed between them. The conditional form of GCA already had been introduced into uGCA paradigm, which also was extended to large-scale network analysis [3, 4]. Then, the derivation process for obtaining the coding length associated its optimal model in uGCA-NML form was illustrated below in detail.

### uGCA-NML—minimax solution for inherent redundancy

Recur to the universal coding, suggested by Kolmogorov, it constructs a code for data sequences such that asymptotically, as the length of data sequence increases, the average code length per symbol would approach the entropy generated the data. Different universal coding schemes thus can be compared in terms of the average code redundancy in its worst-case process, i.e., maximizing the average code length excess over its entropy in the candidate model class. Later on, Clarke and Barron [10, 11], further provided a very accurate asymptotic formula for the code redundancy, defined by a mixture density:

3 $$\begin{aligned} f_{w}(x^{n})= \int f(x^{n}|\theta )\mathrm{d}\omega (\theta ), \end{aligned}$$

namely

4 $$\begin{aligned} E_{\theta }\ln \dfrac{f(x^{n}|\theta )}{f_{w}(x^{n})}=\frac{k}{2}\ln \frac{n}{2\pi e}+\ln \dfrac{|I(\theta )|^{1/2}}{\omega (\theta )}+0(1). \end{aligned}$$

Decades ago, universal coding has evolved into the so-called universal modeling, which is no longer restricted to how to encode data but rather to pursue optimal models, above all an optimal universal model. Distill these thinkings, a universal modeling principle, the MDL for statistical inference, then, generalizes the older idea of parameter estimator in statistics [12–14], and it incorporates the model complexity which affects all aspects of model performance into its coding scheme [8].

Unfortunately, code length within earlier extended coding theorems [15, 16] cannot be sharpened to distinguish by a constant; however, large the data is, and the second term in the right-hand side of Eq. (4) suggests that the constant term can be large indeed when the Fisher information of data generating machinery is nearly singular. Hence, code lengths such as the stochastic complexity would not serve the intended purpose to provide a yardstick, by which model classes can be compared in accordance with a finite and possibly even small amount of data. For this reason, Rissanen pointed that the issues of coding data sequences in a non-redundant procedure [8], should be reconsidered efficiently while paying attention to any potentially large additional terms that may arise.

Among the earlier coding schemes, one stands out as an intuitively appealing candidate for the sought-for code, the so-called maximum-likelihood estimator (MLE) , given by

5 $$\begin{aligned} {\hat{f}}(x^{n})=\dfrac{ f(x^{n}|{\hat{\theta }}(x^{n}))}{\int f(x^{n}|{\hat{\theta }}(x^{n}))\mathrm{d} x^{n}}, \end{aligned}$$

and finite alphabets were also dealt in [17, 18] but without an explicit easy-to-calculate formula. Obviously, for infinite alphabets, the integral domain must be finite for the code to exist. By presenting an implementable version of this coding scheme, in which the maximum-likelihood estimates ${\hat{\theta }}(x^{n})$ are quantized, it had been shown that was equivalent with a two-part code, as discussed in [13], with the inherent redundancy removed. In this case, as long as ${\hat{\theta }}_{n}$ exists for all $x^{n}$, we have

6 $$\begin{aligned} P^{(n)}_{nml}(x^{n})=\dfrac{P_{{\hat{\theta }}_{n}}(x^{n})}{\sum P_{{\hat{\theta }}_{n}}(x^{n})}. \end{aligned}$$

The sequence of distributions $P^{1}_{nml}$, $P^{2}_{nml}$,..., constitutes minimax optimal universal model relative to the considered class ${\mathcal {M}}$, it tries to assign to each $x^{n}$ a probability according to MLE for $x^{n}$ [19]. In addition, the researches were carried further by [6, 8], for sequences $x^{n}$ such that ${\hat{\theta }}(x^{n})\in \Gamma$:

7 $$\begin{aligned} \begin{aligned} L_{n}=-\log f(x^{n}|{\hat{\theta }}(x^{n}))+\frac{k}{2}\ln \frac{n}{2\pi }+\ln \int _{\Gamma }\sqrt{|I(\theta )|}\mathrm{d}\theta +o(1). \end{aligned} \end{aligned}$$

Then, the non-integrability of MLE procedure is the key issue to be solved. However, some of the most important model classes, for example, the class of Gaussian distributions and exponential distributions, are such that the square root of the Fisher information is not integrable nor is the parameter space compact. For these cases, the asymptotic formula Eq. (6) for describing its stochastic complexity term requires a modification, it has been illustrated how such issues can be handled by calculating an asymptotic expression for the stochastic complexity in the all-important Gaussian family, as needed in the regression analysis [8]. As a consequence, in the family of Gaussian distributions, the Fisher information is given by

8 $$\begin{aligned} |I(\beta ,\tau )|=|S|/(2\tau ^{k+2}), \end{aligned}$$

and the integral of its square root dealt by [6, 9] is

9 $$\begin{aligned} \int _{\beta ^{'}S\beta \le R}\int _{\tau _{0}}^{\infty }|I(\beta ,\tau )|^{1/2}d\tau d\beta =(2|S|)^{1/2}\left({\frac{R}{\tau _{0}}}\right)^{k/2}\frac{V_{k}}{k}, \end{aligned}$$

where $V_{k}R^{\frac{k}{2}}=|S|^{-\frac{1}{2}}2(\pi R)^{\frac{k}{2}}/k\Gamma (\frac{k}{2})$ denotes the volume of a *k*-dimensional ball $B=\{\beta ^{'}S\beta \le R\}$. Lower bound $\tau _{0}$ is determined by the precision which the data are written, then ${\hat{\tau }}_{0}=RSS/n$ and ${\hat{R}}=({\hat{\beta }}^{'}X^{'}_{t-k}X_{t-k}{\hat{\beta }})/n$ obtained by MLE. Thus a code length, that is the shortest code length ($L_{X}$ or $L_{X+Y}$), derived from Eq. (7) arrives at

10 $$\begin{aligned} \begin{aligned} { L_{uGCA-NML} }=n\ln \sqrt{2\pi \tau } +\frac{RSS}{2\tau }+\frac{k}{2}\ln \frac{n}{2}-\log \Gamma \left({\frac{k}{2}}\right)+\frac{k}{2}\log \frac{{\hat{R}}}{\tau _{0}}-2\log k . \end{aligned} \end{aligned}$$

### Synthetic experiment protocol

To reveal the specialty of uGCA-NML among several forms, a synthetic network was given by

$$\begin{aligned} \begin{aligned} {\left\{ \begin{array}{ll} x1_{t}=0.68x1_{t-1}-0.24x1_{t-2}+0.45x2_{t-1}-0.15x2_{t-2}+\epsilon _{1}\\ x2_{t}=0.76x2_{t-1}-0.34x2_{t-2}+0.33x1_{t-1}-0.12x1_{t-2}+\epsilon _{2}\\ x3_{t}=0.72x3_{t-1}-0.36x3_{t-2}+0.30x1_{t-1}-0.09x1_{t-2}+\epsilon _{3}\\ x4_{t}=0.68x4_{t-1}-0.22x4_{t-2}+0.42x2_{t-1}-0.19x2_{t-2}+0.33x5_{t-1}-0.14x5_{t-2}+\epsilon _{4}\\ x5_{t}=0.62x5_{t-1}-0.29x5_{t-2}+0.32x2_{t-1}-0.12x2_{t-2}+0.42x4_{t-1}-0.18x4_{t-2}+\epsilon _{5}\\ x6_{t}=0.75x6_{t-1}-0.26x6_{t-2}+0.41x3_{t-1}-0.22x3_{t-2}+0.38x5_{t-1}-0.15x5_{t-2}+\epsilon _{6}. \end{array}\right. } \end{aligned} \end{aligned}$$

**Fig. 1:**
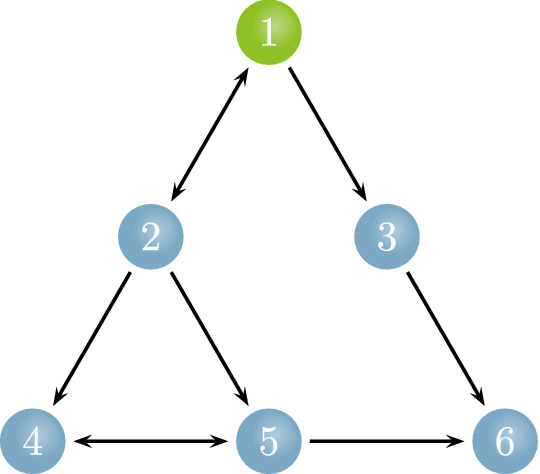
Relationships of simulation data sets in the 6-node networks

Then, several uGCA forms and conventional GCA were compared their characteristics in this synthetic 6-node network, its structural network is presented in Fig. 1. Noise terms $\epsilon _i (i=1, 2,..., 6)$ were Gaussian distribution with mean 0.

### fMRI data within mental arithmetic experiment protocol

In this mental arithmetic experiment, we let ten subjects perform simple one-digit (consisting of 1–10) serial addition (SSA) and complex two-digit (consisting of 1–5) serial addition (CSA) by visual stimulus and simultaneously measured their brain activities with fMRI. Immediately following, each subject was asked to perform the same serial addition arithmetic tasks by an auditory stimulus. Nine right-handed healthy subjects (four female, $24 \pm 1.5$ years old) and one left-handed healthy female subject (24 years old) participated. One of the subject’s(a right-hand male) experimental data was removed due to excessive head motion. All subjects volunteered to participate in this study with the informal written consent by themselves.

## Experiments and results

### Synthetic data

**Fig. 2:**
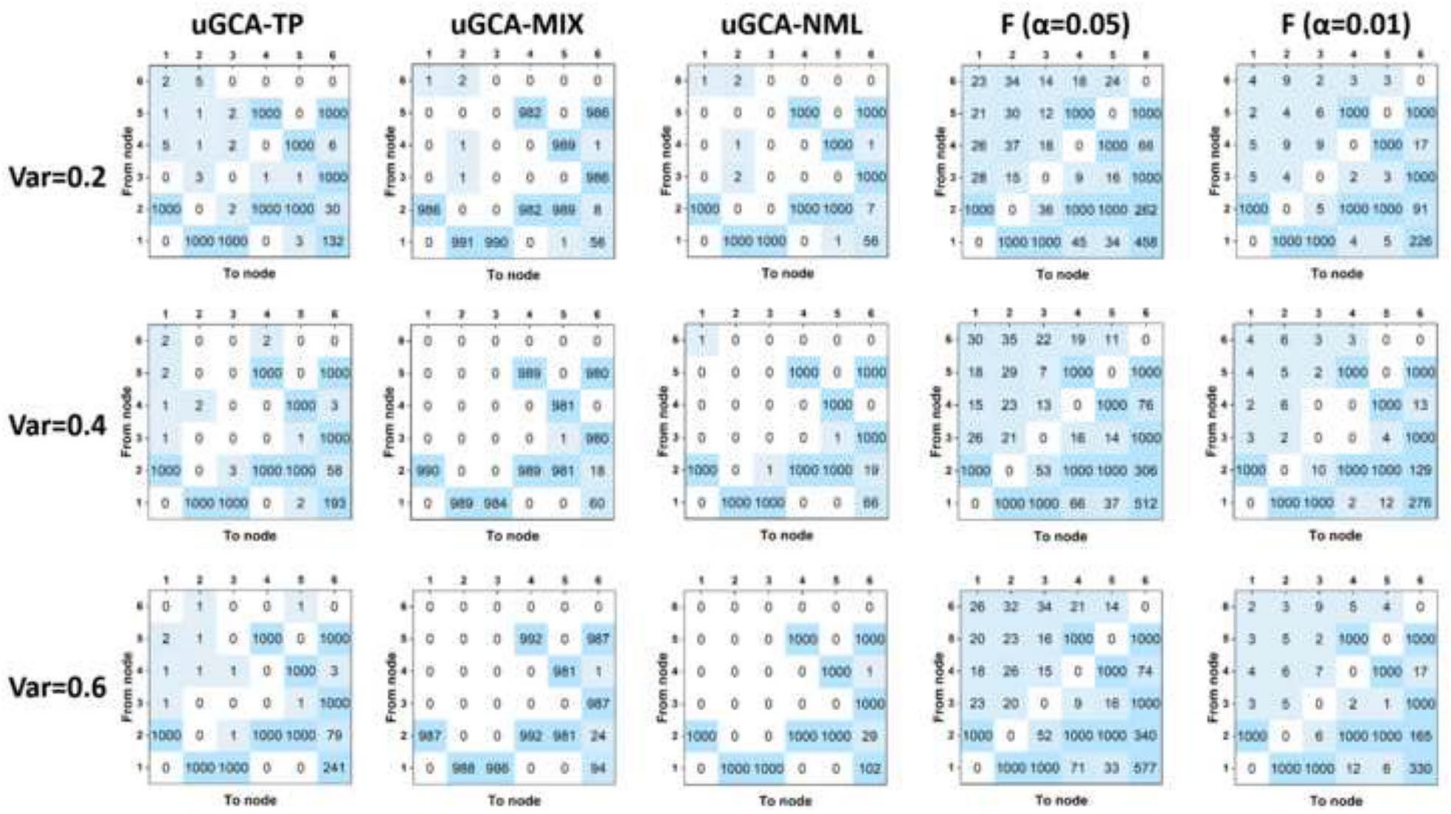
Causal connectivities obtained by several uGCA forms and conventional GCA. Top row represented results in low noise level (var = 0.2), the middle was middle noise level (var = 0.4), the bottom denoted high noise level (var = 0.6). The data length was set to 1000

Figure 2 illustrates causal networks obtained by several uGCA forms and conventional GCA. For true connectivities, except for uGCA-MIX, several uGCA forms and conventional GCA all have an admirable property. As shown in the previous research [4], uGCA-MIX had more chances of producing false negatives because of introducing some priors on estimated parameter distribution. The uGCA-TP and uGCA-NML forms had a very stable identification performance for the true positive rate (TPR). As for false connectivities, the advantages of uGCA paradigm have emerged distinctly. Specifically, uGCA-MIX and uGCA-NML ensured a higher true negative rate (TNR), which meant they both would identify a sparse connection network. Even for uGCA-TP, its false positives also were stifled at a low level. However, poor identification was obvious for conventional GCA in eliminating false connectivities, whatever its confidence level is 0.05 or 0.01. Especially for $1 \rightarrow 6$, $2 \rightarrow 6$, quite a few false positives existed. Although experimental results illustrated that increasing confidence level improved its TNR, the subjectivity of confidence level selection would bring another problem to be dealt with. That is, the ground truth is given in a synthetic data experiment, but in real data, its prior knowledge is usually deficient, which leads to the lack of a uniform yardstick to choose a confidence level. Clearly, the comparisons were presented in Table 1, uGCA-NML obtained higher TNR and TPR, which was less affected by the varied noise. At the same time, uGCA-NML identified the most outstanding ground-truth rate, which conveyed the method’s ability to recognize the real situation more directly and precisely. However, all methods would produce more false connectivities as the noise variance increased, which all were associated with the connectivities $1 \rightarrow 6$, $2 \rightarrow 6$. We consider these increased false connectivities within different noise terms that are due to this specific structural network in Fig. 1 [3, 4]. Generally speaking, uGCA-TP, uGCA-NML, and conventional GCA all had a good anti-interference ability for noise [4]. However, clearly, the uGCA-NML can identify true connectivities with a higher TPR, while ensures higher TNR to eliminate false connections.

**Table. 1:** Comparison between uGCA methods and conventional GCA under different noise level

		uGCA-TP (%)	uGCA-MIX (%)	uGCA-NML (%)	GCA ($\alpha =0.05$) (%)	GCA ($\alpha =0.01$) (%)
Low						
TPR		100	98.678	100	100	100
TNR		99.062	99.662	99.662	94.162	98.010
** Ground-truth rate**		**81.9**	**86.3**	**93.1**	**25.6**	**64.0**
Moderate						
TPR		100	98.478	100	100	100
TNR		98.714	99.662	99.624	93.576	97.729
** Ground-truth rate**		**74.5**	**84.6**	**91.2**	**18.9**	**58.4**
High						
TPR		100	98.678	100	100	100
TNR		98.410	99.433	99.371	93.048	97.148
** Ground-truth rate**		**69.7**	**81.7**	**87.5**	**16.0**	**52.1**

The variance of the low noise level data ranges from 1.5 to 2, and the moderate (high) level data ranges from 3.5 to 3 (5.5–6). The ground-truth rate denoted the total numbers of the obtained individual connection network which was same as the ground-truth network, divided by the sample number (1000)

Bold values indicate more indicative of the method's performance

**Fig. 3:**
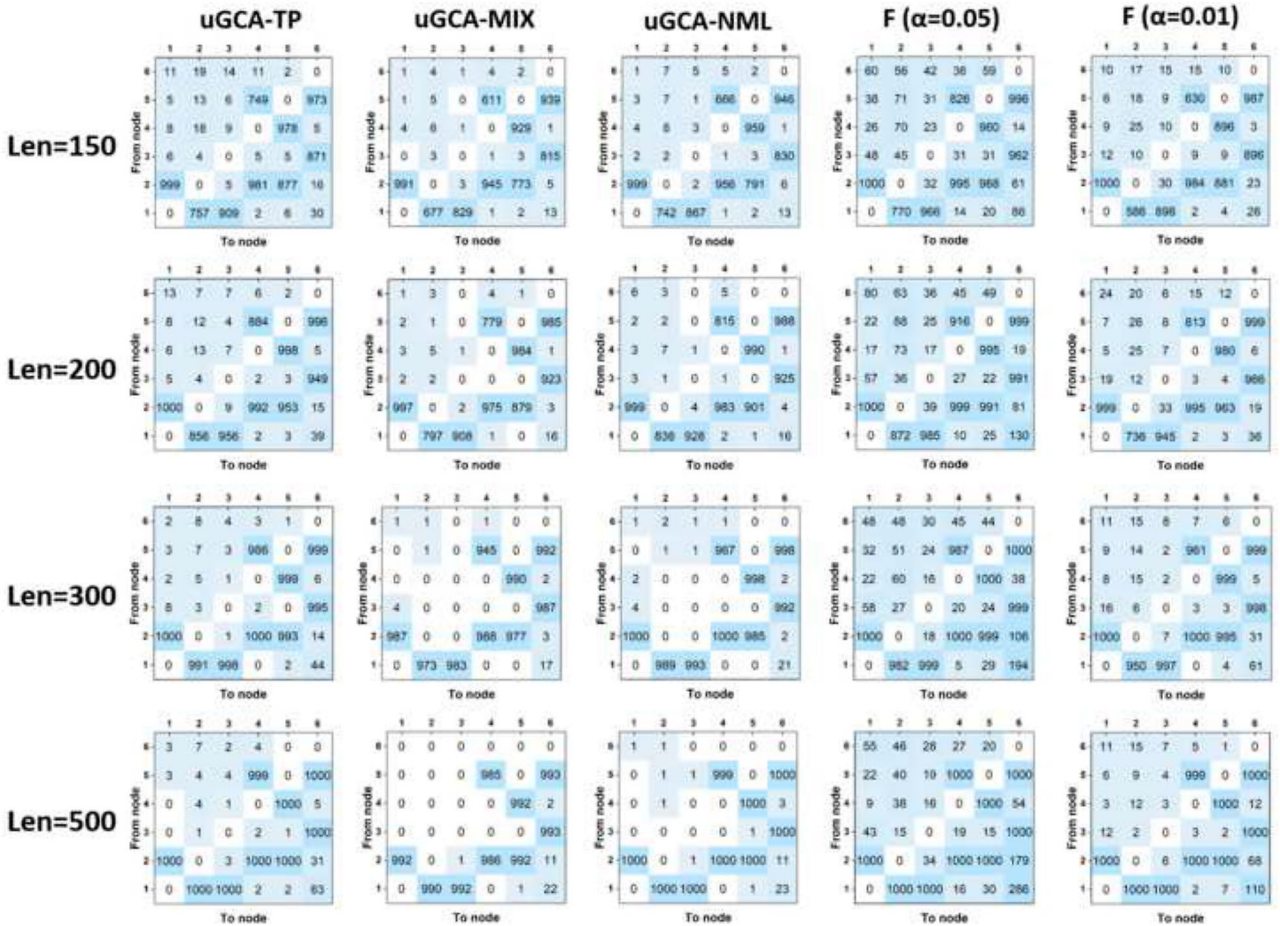
Causal connectivities obtained by uGCA and conventional GCA under different data length. From top row to bottom row, the data length is 150, 200, 300, 500

To further confirm the priority of uGCA-NML, data length was ranged from 150 to 500. For conventional GCA, it identified all true connectivities with high accuracy when data length was above 500, shown in Fig. 3. However, several false connectivities were also increased to a high level when varied data lengths from 200 to 1000, such as $1 \rightarrow 6$, $2 \rightarrow 6$. For uGCA-TP form, it ensured a high TPR when data length was 300. Then varying data length to 500, all true positives were almost fully identified. The uGCA-TP can eliminate false positives as its data length increased, but the false connectivity $1 \rightarrow 6$ had some increase either. As for uGCA-MIX, it obtained a higher accuracy in identifying true negatives within a shorter data length than uGCA-TP. However, uGCA-MIX can not identify the true positives with a high accuracy even data length is 1000. Thus, it stifled false positives to a very low level, which had the highest accuracy in eliminating these spurious connectivities, then identified a very sparse connection network. Similarly, uGCA-NML can ensure good accuracy in identifying true positives as data length was above 300, and almost fully obtained these connectivities when data length was 500. And the direct comparisons illustrated in Table 2, uGCA-NML almost identified a ground truth network in Fig. 1 for every synthetic data sample when data length was 500. On the contrary, other uGCA forms cannot reach the same accuracy when date length was above 300. By the way, these results demonstrated that when data length is below 200, distorted causal networks are identified for both individuals and groups, leading causal investigations unconvincing. And this specific structural network also led to a decline in the accuracy of the ground-truth rate, TPR, and TNR, for which the false connections almost were from $1 \rightarrow 6$, $2 \rightarrow 6$. Therefore, due to the increase of data length, the performance of causal investigation in uGCA-NML had the most obvious improvement. The uGCA-NML seems to rely on long data length to ensure admirable identification ability and is less affected by noise terms. Of course, the uGCA-TP can be regarded as a conservative choice.

**Table. 2:** Comparison between uGCA methods and conventional GCA under different data length

		uGCA-TP (%)	uGCA-MIX (%)	uGCA-NML (%)	GCA ($\alpha =0.05$) (%)	GCA ($\alpha =0.01$) (%)
$$L=150$$						
TPR		89.933	83.433	86.178	93.810	86.200
TNR		99.048	99.710	99.624	95.743	98.705
** Ground-truth rate**		**31**	**17.5**	**22**	**24.4**	**18.5**
$$L=200$$						
TPR		95.378	91.411	92.967	97.200	93.289
TNR		99.181	99.748	99.705	95.610	98.610
** Ground-truth rate**		**56.4**	**41.1**	**48.2**	**29.3**	**40.8**
$$L=300$$						
TPR		99.567	98.022	99.133	99.620	98.878
TNR		99.433	99.857	99.820	98.878	98.890
** Ground-truth rate**		**85.5**	**83.0**	**88.9**	**38.3**	**72.1**
$$L=500$$						
TPR		99.989	99.033	99.989	100	100
TNR		99.324	99.824	99.786	95.186	98.571
** Ground-truth rate**		**86.3**	**91.3**	**95.6**	**34.2**	**72.9**

The data length ranges from 150 to 500, the results ($L=1000$) were present in Table 1, and *L* is the data length

Bold values indicate more indicative of the method's performance

### fMRI data within mental arithmetic experiment

**Fig. 4:**
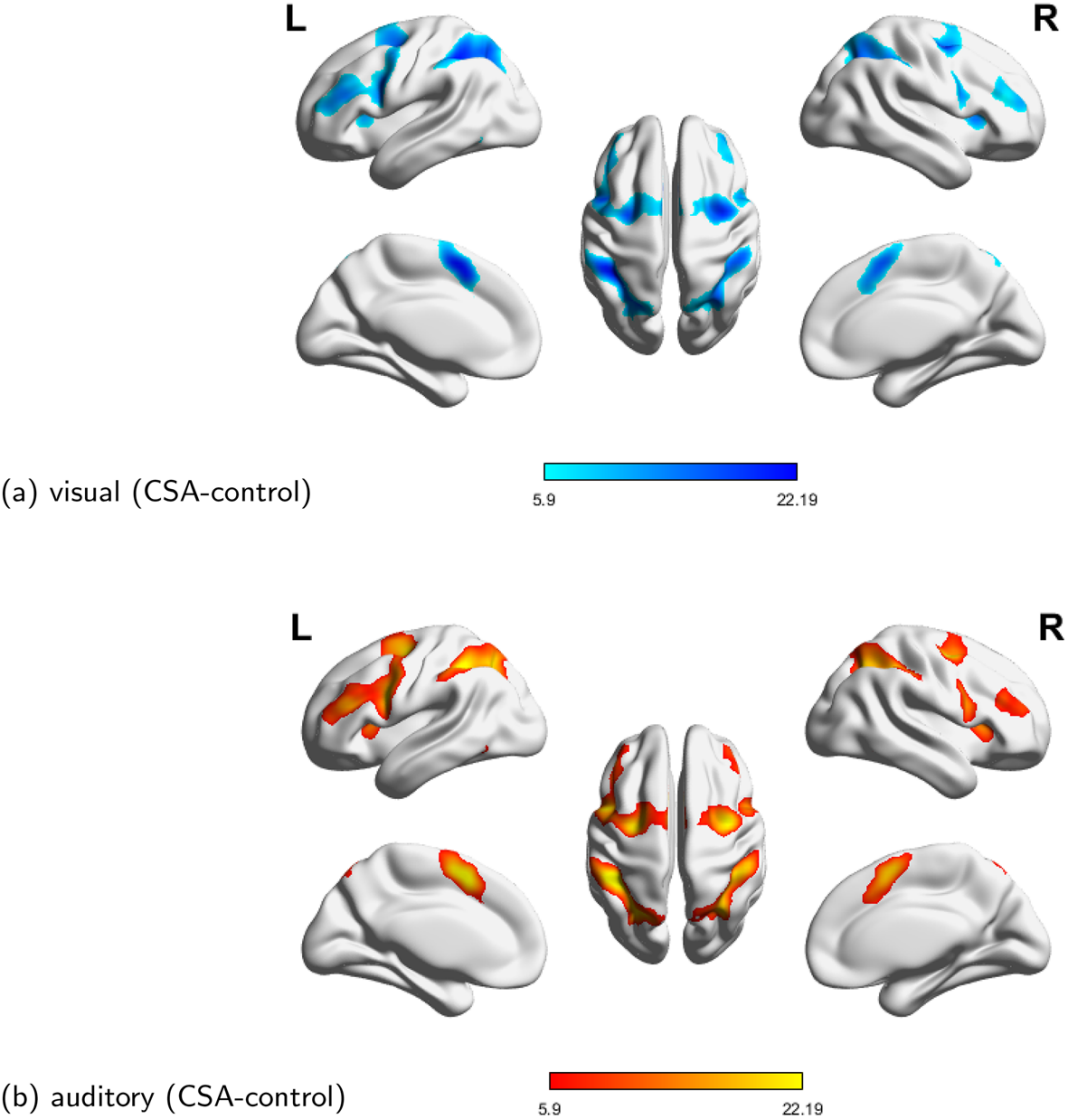
Mental arithmetic of CSA-control state under the two stimuli (visual and auditory), the activation regions were processed by SPM12. **a** CSA-control state under visual stimulus. **b** CSA-control state under auditory stimulus ($P < 0.0001$, uncorrected)

During tasking, these working scenarios of the brain were mental arithmetic tasks, thus these working scenarios can be considered similar regardless of specific stimuli (visual or auditory), respectively. Through the Statistical Parametric Mapping (SPM) software, we can get their mental arithmetic activation regions of the brain, shown in Fig. 4. In these mapped regions through statistical inference, these methods identified causal connectivities of the mental arithmetic network in their own feature space. Then, by comparing their similarities of mental arithmetic networks under different stimuli, we can quantitatively compare their characteristics of several uGCA forms in the causal network of real fMRI data [3, 20].

To compare the similarities among causal networks of different methods, we consider quantifying the mutual information between causal networks under visual and auditory stimulus. Let the joint distribution of two random variables (*X*, *Y*) be *p*(*x*, *y*) , and the marginal distribution be *p*(*x*) , *p*(*y*) , respectively, and the mutual information is the relative entropy of the joint distribution *p*(*x*, *y*) and the marginal distribution *p*(*x*), *p*(*y*), that is

11 $$\begin{aligned} I(X;Y)=\sum _{x\in X}\sum _{y\in Y}p(x,y)\log \frac{p(x,y)}{p(x)p(y)}. \end{aligned}$$

**Fig. 5:**
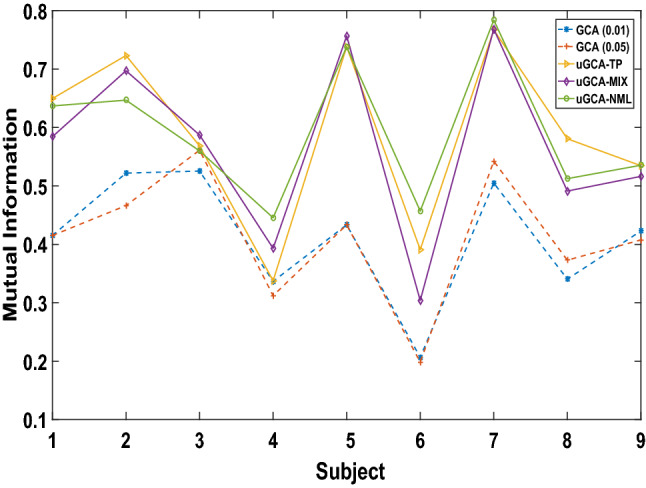
Mutual information of the obtained mental arithmetic networks under two stimuli(visual stimulus and auditory stimulus)

In our mental arithmetic experiment, variable (*X*, *Y*) are the causal networks under visual/auditory, respectively. Intuitively, these two causal networks should be isomorphic mapping, which means their mutual information will maintain a high level. Thus, the priority of different causal investigation methods can be compared by the mutual information between two causal networks, shown in Fig. 5. Clearly, the mutual information of uGCA paradigm revealed that uGCA had a more admirable identification for causal connectivities than conventional GCA whatever the confidence level is. Comparing several form uGCA, their mutual information all held on a high level, and are in good agreement with the simulation results. However, results among these 9 samples illustrated that uGCA-NML obtained a more stable identification level, which demonstrated its priority. In general, uGCA paradigm can ensure a clear superiority over the conventional GCA, and uGCA-NML can be the most recommended choice among these several forms.

**Fig. 6:**
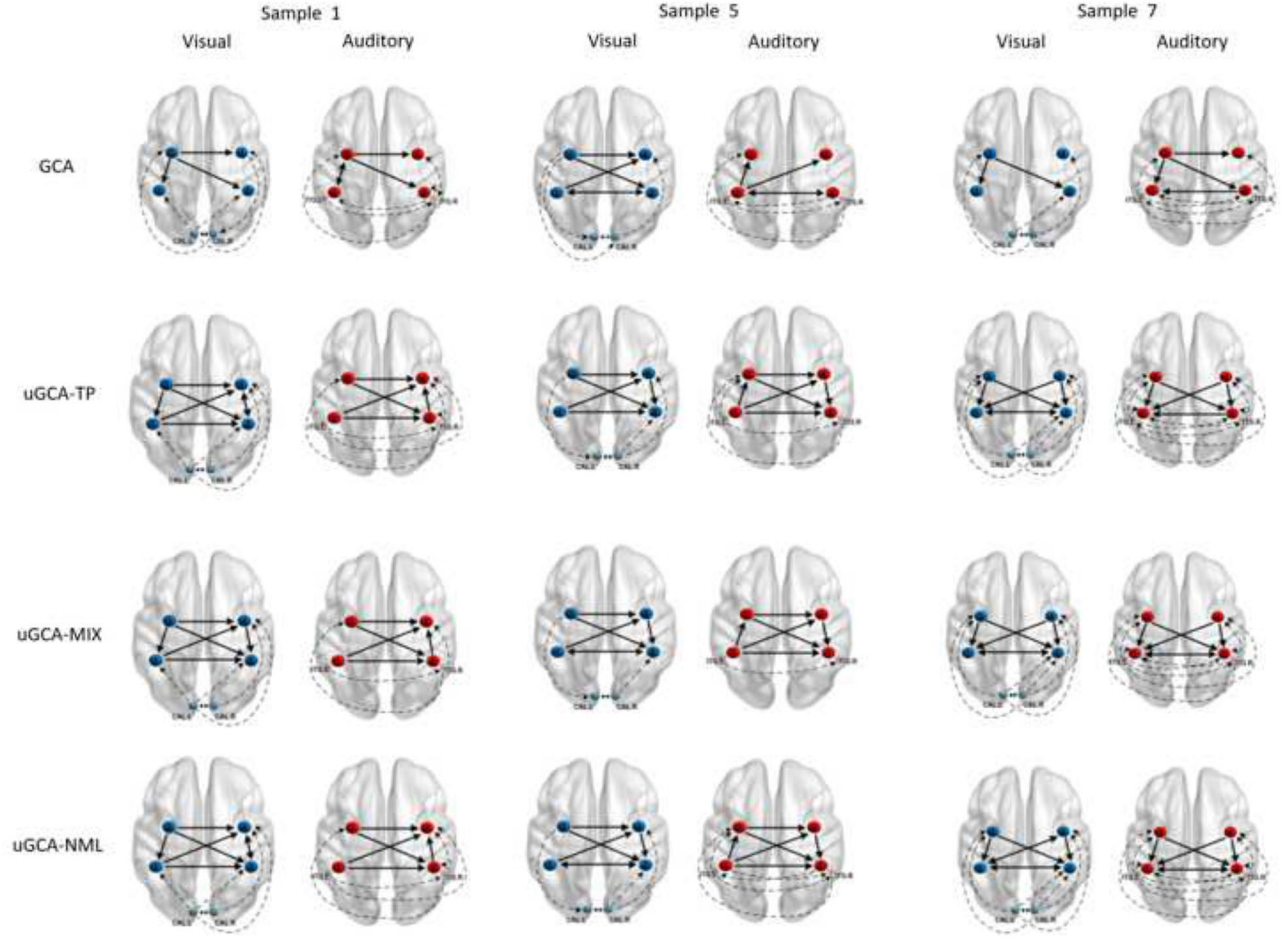
Causal network in the mental arithmetic tasks obtained by uGCA methods and conventional GCA, respectively. With the conventional GCA approach, connected edges of causal networks in two different stimuli were to a large extent distinct. In contrast, for uGCA methods, their connection networks commonly showed high similarities. Node 1, 2, 3, 4 was involving the inside network of mental arithmetic tasks. As for different stimuli input nodes, they were CAL.L, CAL.R, ITG.L, and ITG.R, respectively. The solid lines represent causal connectivities within the mental arithmetic network, and the dashed lines represent causal connectivities involving the input stimulus nodes

To further illustrate this superiority, causal networks obtained by different methods on individuals shown in Fig. 6, respectively. From the mutual information in Fig. 5, uGCA-TP obtained causal networks with the highest similar level in these samples. Clearly, uGCA paradigm identified more similar causal networks between two stimuli than conventional GCA. In these samples, most connecting edges in the mental arithmetic network (containing nodes 1, 2, 3, 4) were identical, only a few edges were different. Even in the whole tasking networks, there only were several different edges in their 6-node networks. In fact, $2^{30}$ possible sub-causal connection networks can be generated in a random 6-node network (only contains 0 and 1). In subject 1, uGCA-TP and uGCA-NML had 7 different edges, uGCA-MIX had 10 different edges. However, as for the driven nodes, uGCA-NML and uGCA-TP obtained a more identical result, which node 4 was the driven node. For subject 5, uGCA-TP had 7 different edges and uGCA-NML had 9 different edges when uGCA-MIX had 5 different edges. Although these, several uGCA forms all obtained an identical driven node, node 2. In subject 8, uGCA-TP and uGCA-MIX only had 3 different edges, uGCA-NML also only had 4 different edges. Obviously, their driven nodes were also identical. In general, these identical mental arithmetic networks obtained through uGCA-MIX showed that the isomorphic mapping phenomenon of three subjects was legible, which meant that the ability of subjects to perform mental arithmetic tasks may be more prominent. Although uGCA-TP had a better performance of similarity measurement in mutual information, uGCA-NML seemed to be more identical in their causal network structure. On the other hand, these results illustrated that uGCA-MIX had a poor anti-interference capability. As mentioned above, uGCA-NML can identify true connections well when eliminating the influence of false connections, then obtain a more sparse connection matrix.

## Discussion

Combining previous and current synthetic data experiments, in this study, we further provided more evidence to demonstrate the priority of uGCA-NML for causal investigation. As we discussed in previous studies, due to some priors employing on the parameter estimation, uGCA-MIX preferred to obtain a sparse causal network, but it may sometimes (in some specific noise level or network architecture) lead to very poor causal identification results because of this over-fitting model selection procedure. As for uGCA-NML, no matter what its noise level was, it can eliminate the influence of false connections better when found real connections, so as to get a sparse connection matrix more accurately. Turn to uGCA-TP, its overall performance may be a compromise between uGCA-MIX and uGCA-NML [3, 4].

In the fMRI experiment, we have demonstrated in previous studies that the mental arithmetic networks obtained by uGCA were more similar, and the isomorphic phenomenon seemed more obvious. Compared with conventional GCA, in which only a few subjects seemed to show clear isomorphism, uGCA integrates the conventional two-stage GCA scheme into a unified framework. And we considered that this isomorphic mapping involving mental arithmetic is a continuous closed process, which requires to keep a consistency of mathematical principles in that quantification process of isomorphism, otherwise, a breakpoint may be brought in. In mathematics, it named a singular point, whose related operations should be closed, otherwise, the processed results may be may deviate from the original space and become very distorted. A widely accepted view states that the original model space of generating the data set can not be found at all. Thus, toward the length of coding model complexity, several uGCA forms provided different solutions, which mapping the descriptive model into different feature spaces to approach the original model space in different aspects. With the help of mutual information, we further compared several uGCA forms and conventional GCA. The uGCA paradigm had a clear priority over conventional GCA. Then, among these forms, uGCA-NML obtained a more stable result, while it ensured accurate causal networks, which identified high-level similarities of causal connectivities. By the way, uGCA-TP also obtained nearly identical connection networks under visual/auditory stimuli, and uGCA identified some acceptable results either. Adopting a crude two-part coding version, uGCA-TP benefits from this parsimony coding scheme, it will also have some advantages in real fMRI data.

To sum up, uGCA-NML has certain preferential selectivity among these forms. Compared with uGCA-TP, it eliminates the inherent redundancy of model parameter estimation, while compared with uGAC-MIX, it does not require a prior and has a more stable causal identification. Moreover, these results indicated that causal isomorphism does exist during mental arithmetic tasks. Actually, the postulation that the isomorphic mapping of the brain under similar tasks is not fabricated from the single experimental phenomena. Gradually over the years, some researchers have tried to demonstrate this capability that the brain perceives our world by the analogical reasoning [21–27]. And some other researchers also suggested using category theory to mathematically demonstrate how analogical reasoning in the human brain get rid of the spurious inferences that puzzle traditional artificial intelligence modeling (called systematicness) [28–30]. As a consequence, a more unified causal investigation method, uGCA-NML, will more appropriate for the brain with such logical rigor.

## Conclusion

The uGCA paradigm first maps the original space into a unified code length guided space, and then to identify the causal connectivities. Therefore, this allows data sets to hold their original correlations as much as possible, thus obtaining an optimal approximate description for their correlations in the original space. Actually, different uGCA forms provided different aspects to approach the ground truth, and obtained the optimal descriptive model in their own characteristic spaces. In this paper, we conclude a standpoint that uGCA-NML owns a priority among these several uGCA forms. Although several uGCA forms have their own different advantages, especially for this kind of exploratory study of causal investigation, the comparison of different methods is still controversial. However, for causal investigation in our unified code length guided framework, uGCA-NML will be the most recommended choice.

## Data Availability

Data is available from the corresponding author upon request.
